# Microglia-derived TNF-α mediates endothelial necroptosis aggravating blood brain–barrier disruption after ischemic stroke

**DOI:** 10.1038/s41419-019-1716-9

**Published:** 2019-06-20

**Authors:** An-Qi Chen, Zhi Fang, Xiao-Lu Chen, Shuai Yang, Yi-Fan Zhou, Ling Mao, Yuan-Peng Xia, Hui-Juan Jin, Ya-Nan Li, Ming-Feng You, Xu-Xia Wang, Hao Lei, Quan-Wei He, Bo Hu

**Affiliations:** 1 Department of Neurology, Union Hospital, Tongji Medical College, Huazhong University of Science and Technology, Wuhan, China; 2 National Center for Magnetic Resonance in Wuhan, State Key Laboratory of Magnetic Resonance and Atomic and Molecular Physics, Wuhan Institute of Physics and Mathematics, Chinese Academy of Sciences, Wuhan, China

**Keywords:** Blood-brain barrier, Stroke

## Abstract

Endothelium (EC) is a key component of blood–brain barrier (BBB), and has an important position in the neurovascular unit. Its dysfunction and death after cerebral ischemic/reperfusion (I/R) injury not only promote evolution of neuroinflammation and brain edema, but also increase the risk of intracerebral hemorrhage of thrombolytic therapies. However, the mechanism and specific interventions of EC death after I/R injury are poorly understood. Here we showed that necroptosis was a mechanism underlying EC death, which promoted BBB breakdown after I/R injury. Treatment of rats with receptor interacting protein kinase 1 (RIPK1)-inhibitor, necrostatin-1 reduced endothelial necroptosis and BBB leakage. We furthermore showed that perivascular M1-like microglia-induced endothelial necroptosis leading to BBB disruption requires tumor necrosis factor-α (TNF-α) secreted by M1 type microglia and its receptor, TNF receptor 1 (TNFR1), on endothelium as the primary mediators of these effects. More importantly, anti-TNFα (infliximab, a potent clinically used drug) treatment significantly ameliorate endothelial necroptosis, BBB destruction and improve stroke outcomes. Our data identify a previously unexplored role for endothelial necroptosis in BBB disruption and suggest infliximab might serve as a potential drug for stroke therapy.

## Introduction

Ischemic stroke is a major cause of death and disability in the world^1^. Destruction of blood–brain barrier (BBB) after ischemic stroke is an intractable event that conduces to further progress and enlargement of the injury^2,3^. Evidence has accumulated that death of Endothelium (EC) contributes to vascular injury and BBB disruption after I/R injury^4^. However, it remains unclear the precise mechanism of EC death after ischemic/reperfusion (I/R) injury and whether specific interventions of EC death significantly ameliorate BBB leakage. Therefore, how to protect BBB integrity and function by preventing EC death is potential important drug targets for future ischemic stroke therapies.

It is well established that cell death includes two kinds of pathway: apoptosis and necrosis. Apoptosis is a programmed form of cell death, whereas necrosis is a passive cell death mainly caused by overwhelming inflammation or injury stress and cannot be effectively interfered^5^. Thus, recently more researchers embrace the concept of the necroptosis, a caspase-independent programmed form of necrosis. Necroptosis is morphologically characterized by cellular organelle swelling and cell membrane rupture that is mediated by death signal pathways^6^. Proinflammatory cytokines in inflammatory microenvironment, such as tumor necrosis factor-α (TNF-α), depending on their receptors, such as TNF receptor 1 (TNFR1), induce necrosome formation of receptor-interacting protein kinase 1 (RIPK1) and RIPK3, which result in phosphorylation and oligomerization of MLKL^7,8^. Accordingly, the inflammation-induced necroptosis activation served as a vital role in pathogenesis of many diseases, including neurodegeneration^9^, cancer^10^ and autoimmune disease^11^. Moreover, necroptosis plays an important effect on ischemic disease as well. Inhibition of programmed necrosis can alleviate acute kidney ischemia-reperfusion injury^12^, improve ventricular remodeling after myocardial infarction^13^, reduce neuron loss in ischemic stroke^14^ and so on. However, the involvement of necroptosis in the pathogenesis of microvascular EC death after I/R injury and how to maintain BBB integrity by interfering the TNF-α mediated necroptosis are still unclear.

Inflammation is currently considered as a promising target for the development of new stroke therapies^15^. Microglia, the main immune cells of the brain, responds to cerebral I/R injury by becoming activated and developing into classic M1-like (proinflammatory) or alternative M2-like (anti-inflammatory) phenotypes^16^.

There is a currently debatable point about the contribution of activated microglia to neuronal death and stroke progression. Previous studies suggested that perivascular microglia contributed to BBB breakdown and disintegration in ischemic stroke^17^. However, it is also unclear whether perivascular activated microglia such as M1-like microglia could play a role in EC death, one of most fundamental process of the collapse of BBB after I/R injury. In this study, we found a previously unexplored role of M1-like microglia-induced endothelial necroptosis in BBB disruption and suggested infliximab might serve as a potential drug for stroke therapy.

## Results

### EC necroptosis is activated after stroke

**Fig. 1:**
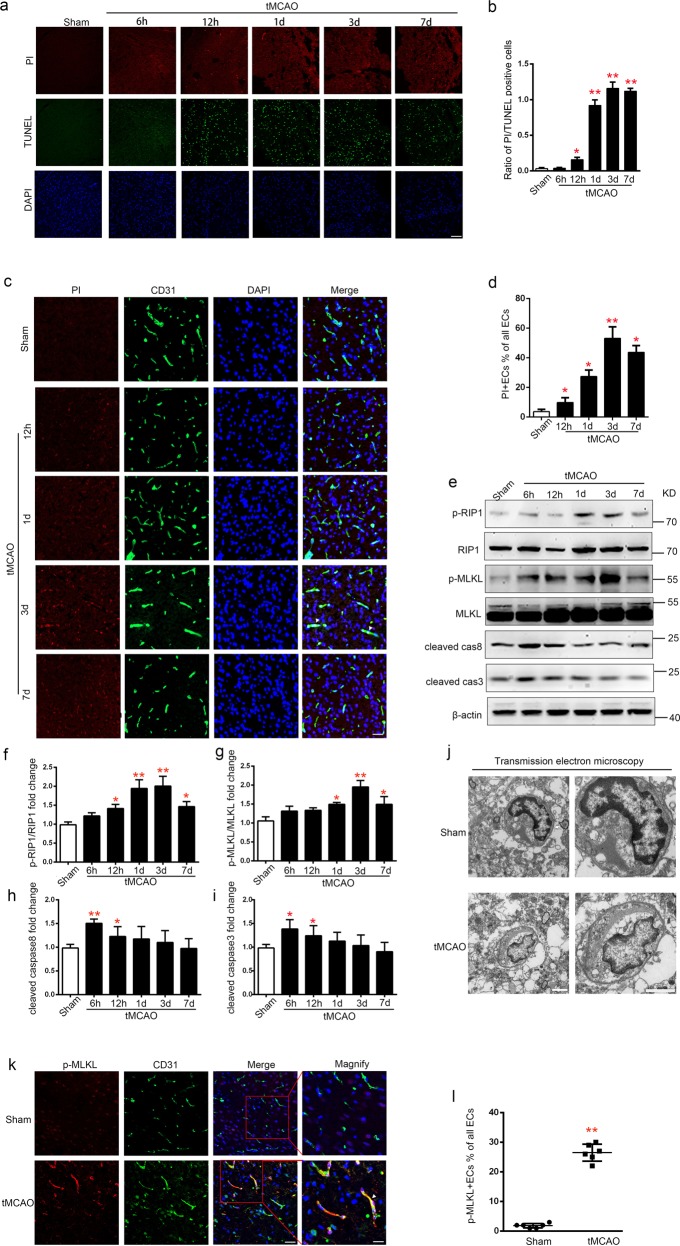
Necroptosis activated after ischemic stroke in Endothelium. **a**, **b** Representative images and statistical results of PI (red, represents necrosis)/TUNEL (green, represents apoptosis) staining of cerebral cortex at 6 h, 12 h, 1 day, 3 day, and 7 day after tMCAO. *n* = 5; *P *<* 0.01 vs. Sham group, **P *<* 0.0001 vs. Sham group; Scale bar: 100 μm. **c**, **d** Representative images and statistical results of CD31 (green) and PI (red) staining of rat brain sections from Sham and tMCAO (12 h, 1 day, 3 day, 7 day) groups. Arrows indicate the colocalization of CD31 and PI. *n* = 6; *P *<* 0.01 vs. Sham group, **P *<* 0.0001 vs. Sham group. Scale bar: 50 μm. **e**–**i** Expression levels of p-RIP1/RIP1, p-MLKL/MLKL, Casp 3 and Casp 8 in lateral cortex tissues of the peri-infarct area after tMCAO (3 day). *n* = 6; *P *<* 0.05 vs. Sham group; **P < 0.01 vs. Sham group. **j** Representative electron microscopy images of EC from ischemic cortex after tMCAO (3 day). EC from ischemic cortex exhibited translucent cytoplasm, swelling mitochondria, and membrane breakdown. *n* = 3; Scale bar: 2 μm (merged pictures) and 1 μm (magnified pictures). **k**, **i** Representative images and statistical results of CD31 (green) and p-MLKL (red) staining of rat brain sections from Sham and tMCAO (3 day) groups. Arrows indicate the colocalization of CD31 and p-MLKL. Scale bar: 100 μm (merged pictures) and 50 μm (magnified pictures); *n* = 6; tMCAO group 26.50 ± 1.176% vs. Sham group 1.833 ± 0.3073%; **P *<* 0.001 (All values from three to six independent experiments are presented as mean ± S.E.M.)

To explore the manner of cell death in brain tissue after stroke, we determined the number of propidium iodide-positive (PI+) and TdT-mediated dUTP nick end labelling (TUNEL+) cells in ischemic cortex at 6 h, 12 h, 1 day, 3 day, and 7 day after transient middle cerebral artery occlusion (tMCAO) (Fig. 1a). After ischemic injury, the ratio of PI+/TUNEL+ cells gradually increased from 12 h to 3 day and remained constant at 7 day in the periinfarct area after tMCAO (Fig. 1b). Immunofluorescent staining showed that the ratio of PI + EC in ischemic cortex started to arise from 12 h, peaked on the 3rd day after tMCAO. (Fig. 1c, d). In parallel, western blotting analysis of lysis extracted from lateral cortex tissues of peri-infarct area showed that the expression levels of p-RIP1/RIP1 and p-MLKL/MLKL, hallmark of necroptosis activation, progressively increased from the 1st day and reached the highest levels on the 3rd day, however decreased on the 7th day after tMCAO. Activation of apoptosis, represented by cleaved caspase 3 (Casp3) and cleaved caspase 8 (Casp8)^18^, increased within 12 h and then decreased from the 1st day to the 7th day after tMCAO (Fig. 1e–i). Thus, we chose the 3rd day after tMCAO as a main time point for underlying mechanism research of EC necroptosis in vivo. The electron microscopy image of periinfarct area showed that necroptosis of vascular EC was activated on the 3rd day after tMCAO (Fig. 1j). p-MLKL (a marker of necroptosis)^10^ and CD31 (a marker of EC) double-labeled cells in ischemic cortex also showed the same trend as that revealed with electron microscope and western blotting analyses (Fig. 1k, l). Together, these findings suggest that necroptosis of vascular EC was activated in ischemic cortex after stroke.

### EC necroptosis increases BBB permeability after tMCAO

**Fig. 2:**
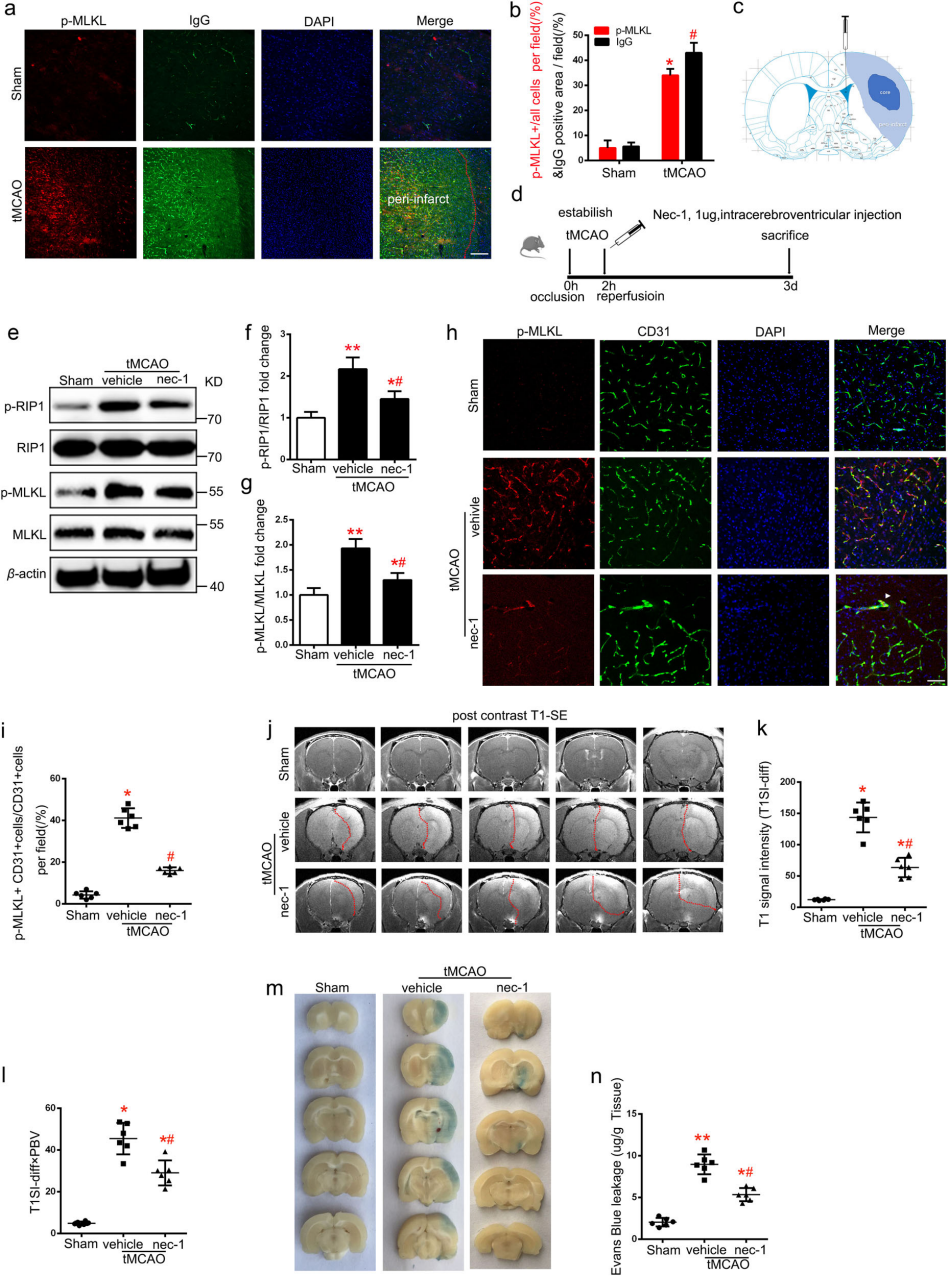
Inhibition of necroptosis decreases BBB disruption after ischemia reperfusion. **a**, **b** Representative images and statistical results of IgG (green, represents BBB leakage) and p-MLKL (red) staining of rat brain sections from Sham and tMCAO (3 day) groups. *n* = 3; *P < 0.005 as of p-MLKL in tMCAO group vs. Sham group; ^#^*P* < 0.001 as of IgG in tMCAO group vs. Sham group; Scale bar: 100 μm. **c** Schematic diagram showing the lateral cerebral ventricle injection area in rat brain. **d** Experimental scheme. **e–g** Expression levels of p-RIP1/RIP1 and p-MLKL/MLKL proteins in ischemic lateral cortex after tMCAO (3 day). *n* = 6; *P < 0.005 vs. Sham group; **P < 0.0001 vs. Sham group; ^#^*P* < 0.001 vs. vehicle group. **h**, **i** Representative images and statistical results of CD31 (green) and p-MLKL (red) staining of rat brain sections from tMCAO (3 day) and Nec-1-injected groups. Arrows indicate the colocalization of CD31 and p-MLKL. *n* = 6; vehicle group 41.17 ± 1.922 % vs. Sham group 4.33 ± 0.7149 %; *P < 0.001; Nec-1 injected group 41.17 ± 1.922 % vs. vehicle group 16.00 ± 0.6325 %; ^#^*P* < 0.001; Scale bar: 100 μm. **j–l** Representative MRI post-contrast T1-SE images of rat brains from Sham, vehicle, and Nec-1-injected groups. BBB permeability was represented by T1SI-diff and T1SI-diff × PBV. *n* = 6; *P < 0.0001 vs. Sham group; ^#^*P* < 0.005 vs. vehicle group; ^##^*P* < 0.001 vs. vehicle group. **m**, **n** Evans blue leakage of rat brains in coronal sections (**m**) and extravasation (μg/g tissue) (**n**) from Sham, vehicle, and Nec-1-injected groups. *n* = 6; *P < 0.001 vs. Sham group; **P < 0.0001 vs. Sham group; ^#^*P* < 0.001 vs. vehicle group

To explore the relationship between EC necroptosis and BBB permeability, we conducted immunoglobulin G (IgG) immunofluorescent staining that could indicate BBB disruption after tMCAO^19^. Compared to the sham group, p-MLKL staining and IgG staining of the ischemic cortex (Fig. 2a) showed a similar trend, indicating that activation of necroptosis was accompanied with BBB disruption after tMCAO (Fig. 2b). Necrostatin-1(Nec-1), a necroptosis inhibitor, was injected into the lateral ventricles of right ischemic cortex after establishment of tMCAO (Fig. 2c, d)^14^. Treatment with Nec-1 decreased p-RIP1/RIP1 and p-MLKL/MLKL protein expression ratio in ischemic cortex on the 3rd day after tMCAO (Fig. 2e–g). Necroptosis of EC, indicated by p-MLKL and CD31 staining, also showed similar results to those of western blotting. These findings indicated that necroptosis of ECs was inhibited by Nec-1 after tMCAO (Fig. 2h, i). Magnetic resonance imaging (MRI) studies were performed to further confirm the in vivo protective effects of Nec-1 against BBB disruption after tMCAO. After intracerebroventricular injection of Nec-1 (1 µg) after tMCAO, the quantification T1SI-diff and T1SI-diff × PBV, which reflect BBB permeability, revealed a marked reduction compared with the vehicle group on the 3rd day after tMCAO (Fig. 2j–l). Analysis of Evans blue extravasation was consistent with MRI results (Fig. 2m, n). These results suggest that inhibition of endothelial necroptosis has a protective effect against vascular permeability and BBB breakdown.

### Microglia induces EC necroptosis after I/R injury

**Fig. 3:**
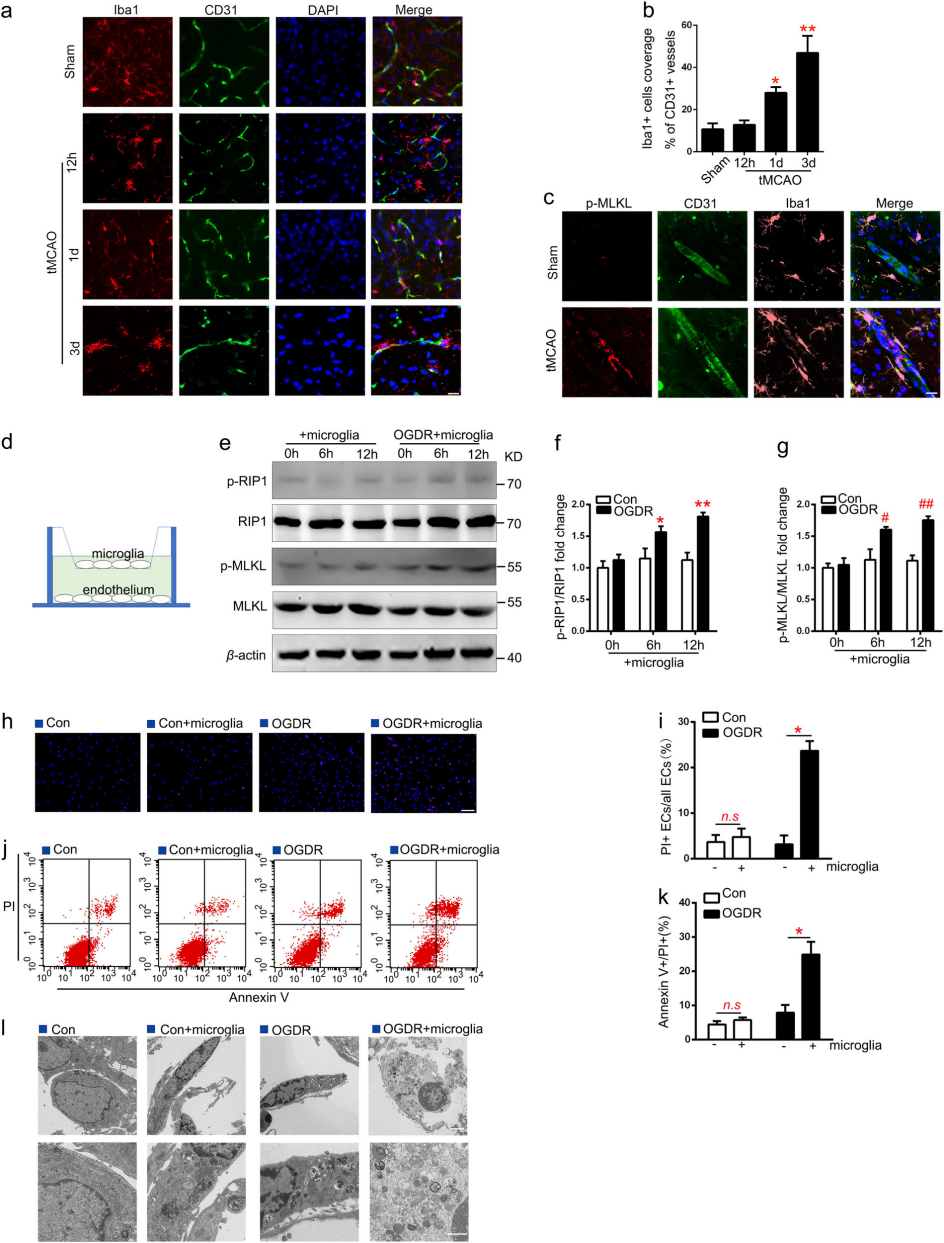
Necroptosis of ECs increases when co-cultured with microglial under OGDR. **a**, **b** Representative images and statistical results of Iba1+(red) cells coverage on CD31+ cells (green) in the peri-infarct area at 12 h, 1 and 3 day after tMCAO. *n* = 6; *P < 0.01 vs. Sham group; **P < 0.005 vs. Sham group; Scale bar: 20 μm. **c** Representative images of CD31 (green), Iba1(pink), and p-MLKL (red) staining of rat brain sections from Sham and tMCAO (3 day) groups. *n* = 3; scale bar: 20 μm. **d** Illustration of the in vitro co-culture system. **e–g** Expression levels of p-RIP1/RIP1 and p-MLKL/MLKL in ECs under different conditions. *n* = 6; *P < 0.05 vs. control 6 h group; **P < 0.001 vs. control 12 h group; ^#^*P* < 0.01 vs. control 6 h group; ^##^*P* < 0.001 vs. control 12 h group. **h**, **i** Representative images of PI (red) and Hoechst (blue) of ECs (solo-cultured or co-cultured with microglia) subjected to normal condition (con) or 12 h OGDR. *n* = 3; *P = 0.0013 vs. solo-culture under OGDR conditions; Scale bar: 100 μm. **j–k** Representative flow cytometry images of ECs stained with PI/annexin V. The necroptosis activation was represented by ratio of PI + /annexin V + . *n* = 3; *P = 0.0091 vs. solo-culture under OGDR. **l** Representative electron microscopy images of ECs. ECs co-cultured with microglia under OGDR exhibited translucent cytoplasm, swelling mitochondria, and membrane disruption. *n* = 3; Scale bar: 2 μm (merged pictures) and 1 μm (magnified pictures)

To investigate the mechanisms underlying EC necroptosis after I/R injury, expression of p-RIP1/RIP1 and p-MLKL/MLKL in EC were assessed at 2, 4, 8, and 12 h after oxygen glucose deprivation/reoxygenation (OGDR) (Supplementary Fig. 1a). Western blotting showed that the difference of protein expression levels of p-RIP1/RIP1 and p-MLKL/MLKL was not statistically significant (Supplementary Fig. 1b and c). As previous studies have reported, microglia secrete a large number of inflammatory factors that may induce necroptosis^20^ and perivascular microglia contributed to BBB breakdown and disintegration in ischemic stroke^17^. We further analyzed the number of microglia contacting vessels by double-staining with microglia marker Iba1 and CD31. The number of microglia in the periphery of penumbral microvessels increased in a time-dependent manner from 12 h to 3rd day after tMCAO (Fig. 3a, b). And we found a close association of active microglia with p-MLKL-positive EC in the ischemic border zone on 3rd day after tMCAO (Fig. 3c). These results revealed a positive association between microglia accumulation and EC necroptosis in the periinfarct area after tMCAO. An in vitro co-culture system of primary rat EC and microglia cells was established to mimic ischemia-reperfusion injury in vitro, as shown in Fig. 3d, microglia cells were seeded on a membrane (0.4 µm pore, Corning Transwell culture plate, USA) in the upper chamber and ECs were seeded on the substratum culture plate. The expression levels of p-RIP1/RIP1 and p-MLKL/MLKL protein in EC were determined using western blotting (Fig. 3e). Compared to the normal co-culture group, the ratios of p-RIP1/RIP1 and p-MLKL/MLKL of EC were up-regulated in a time-dependent manner after OGDR (Fig. 3f, g). Then, ECs (solo-cultured or co-cultured with microglia) were subjected to normal conditions or OGDR for 12 h, respectively. PI/Hoechst staining showed that, compared to the solo-culture group, the number of PI + EC in the co-culture system significantly increased after OGDR (Fig. 3h, i), indicating that endothelial necroptosis is activated by microglia after OGDR. In addition, annexin V/PI flow cytometry (Fig. 3j, k) results showed a similar trend to that of the PI/Hoechst staining analysis. Transmission electron microscopy results were also consistent with our findings, in which EC co-cultured with microglia after OGDR treatment exhibited morphological characteristics typical of necroptosis, characterized by translucent cytoplasm, mitochondrial swelling, enlarged cell volume, and membrane disruption (Fig. 3l).

### M1-like microglia induces EC necroptosis by secreting TNF-α

**Fig. 4:**
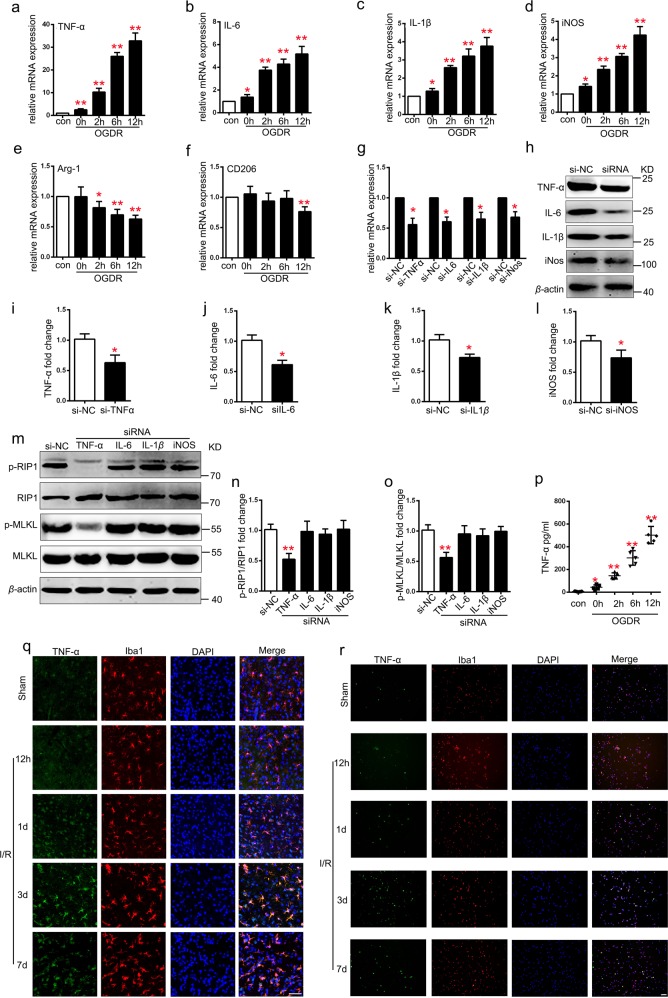
Knockdown of TNF-α in M1 type microglia reduces EC necroptosis **in** co-culture system under OGDR. **a–f** mRNA expression of M1 makers (TNF-α, IL-1β, iNOS, and IL-6) and M2 makers (Arg-1 and CD206) in microglia subjected to OGDR (0, 2, 6, and 12 h). *n* = 6; *P *<* 0.005 vs. control group; **P < 0.0001 vs. control group. **g–l** Expression levels of TNF-α, IL-1β, iNOS, and IL-6 protein in microglia pretreated with corresponding siRNAs. *n* = 6; *P < 0.001 vs. si-NC group; **P < 0.0001 vs. si-NC group. **m–o** Expression of p-RIP1/RIP1 and p-MLKL/MLKL in ECs co-cultured with different siRNA pretreated microglia under 12 h OGDR. *n* = 6; **P < 0.0001 vs. si-NC group. **p** ELISA analysis of TNF-α concentration in supernatants of microglia subjected to OGDR for 0, 2, 6, and 12 h. *n* = 6; *P *<* 0.005 vs. control group; **P *<* 0.0001 vs. control group. **q** Representative images of TNF-α (green) and Iba1(red) staining of rat brain sections from sham and tMCAO (12 h, 1 day, 3 day) groups. *n* = 3; scale bar: 100 μm. **r** Representative images of TNF-α (green) and Iba1(red) staining of microglia acutely isolated from peri-infarct area in sham and tMCAO (12 h, 1 day, 3 day). *n* = 3; scale bar: 100 μm

The expression of proinflammatory cytokines was detected using real-time PCR (RT-PCR) to research the phenotype of microglia under OGDR. The mRNA expression levels of M1 phenotype related proinflammatory molecules (TNF-α, IL-1β, iNOS, and IL-6) increased in a time-dependent manner after OGDR (Fig. 4a–d). In contrast, expression levels of the M2 phenotype associated inflammatory molecules (Arg-1 and CD206)^21^ significantly declined after OGDR (Fig. 4e, f). To further explore the mechanism of the M1 phenotype microglia-mediated induction of endothelial necroptosis after OGDR, we used small interfering RNAs (siRNAs) to knockdown the expression of genes of the proinflammatory molecules secreted by microglia after OGDR. Microglia was transduced with siRNA for TNF-α, IL-1β, iNOS, and IL-6, and RT-PCR and western blotting results indicated a successful gene editing (Fig. 4g–l). Compared to the vehicle-siRNA (Si-NC) group, knockdown of TNF-α in microglia significantly reduced the expression levels of endothelial p-RIP1/RIP1 and p-MLKL/MLKL in a co-culture system after OGDR. In contrast, the expression levels of these proteins barely changed after knockdown of IL-1β, iNOS, and IL-6 in microglia (Fig. 4m–o). In addition, microglia-secreted TNF-α detected by ELISA was up-regulated after OGDR in a time-dependent manner (Fig. 4p). Migration tests show that TNF-α recruited microglia in a dose-dependent manner (Supplementary Fig. 2a, b). Double staining of rat brain tissue sections with TNF-α and Iba1 suggested that TNF-α was mainly expressed on the microglia, increased from 12 h and reached highest levels on the 3rd day after tMCAO (Fig. 4q). Moreover, the expression levels of TNF-α in acutely isolated microglia after tMCAO was consistent with the double staining results of rat brain tissue sections (Fig. 4r). These findings suggest that M1 phenotype microglia-secreted TNF-α leads to necroptosis of EC after tMCAO.

### Inhibition of NF-κB nuclear translocation decreases microglia-derived TNF-α-induced endothelial necroptosis under OGDR

**Fig. 5:**
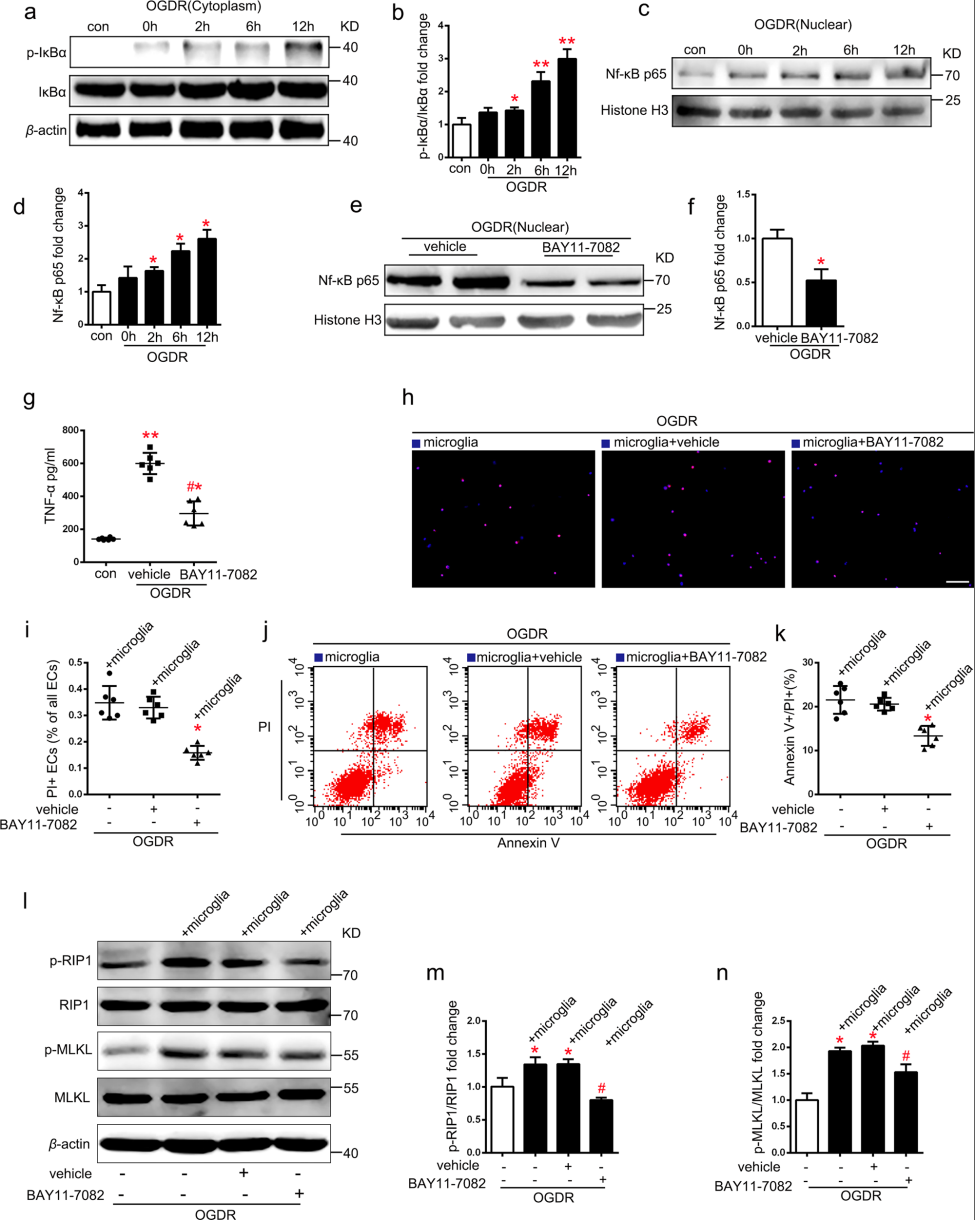
Reversed nuclear localization of NF-kB (P65) in microglia reduces microglia TNF-α expression and inhibits EC necroptosis in co-culture system under OGDR. **a**, **d** Expression levels of p-IκBα/IκBα in cytoplasm (**a**, **b**) and NF-kB (p65) nuclear (**c**, **d**) from microglia subjected to 0, 2, 6, and 12 h OGDR. *n* = 6; *P *<* 0.05 vs. control group; **P *<* 0.01 vs. control group. **e–f** Expression levels of NF-κB (p65) in microglia pretreated with or without BAY-117082 under OGDR. *n* = 6; *P = 0.0069 vs. vehicle group. **g** ELISA analysis for TNF-α show that BAY-117082 reduced TNF-α secretion from microglia under OGDR. *n* = 6; *P < 0.001 vs. control group; **P < 0.0001 vs. control group; ^#^*P* < 0.0001 vs. vehicle group. **h–k** Representative images and statistical results of PI (red)/Hoechst (blue) staining and PI/annexin V flow cytometry of ECs treated with microglia (pretreated with BAY-117082 or vehicle) in co-culture system under OGDR. *n* = 6; *P *<* 0.01 vs. vehicle group; Scale bar: 100 μm. **l–n** Expression levels of p-RIP1/RIP1 and p-MLKL/MLKL in ECs from different group. *n* = 3; *P < 0.001 vs. control group; ^#^*P* < 0.01 vs. vehicle group

To investigate the upstream mechanism of microglia activation, cytosol and nuclear extracts were obtained 0, 2, 6, and 12 h after OGDR (Figs. 3c and 5a). Western blotting analysis showed that p-IκBα/ IκBα expression levels in cytosol extracts from microglia gradually increased after OGDR (Fig. 5b), and NF-κB (p65) protein expression levels in nuclear extracts also increased after OGDR. (Fig. 5d). Treatment with the NF-κB inhibitor (BAY11-7082) resulted in a reduction of approximately 50 % in NF-κB nuclear translocation in microglia subjected to OGDR (Fig. 5e, f). Furthermore, the inhibition of NF-κB nuclear translocation also decreased TNF-α release from microglia after OGDR (Fig. 5g). To further verify the role of microglia NF-κB inhibition on endothelial necroptosis after OGDR, microglia were treated with BAY11-7082 for 30 min and then co-cultured with ECs under OGDR. EC were stained with PI and Hoechst (Fig. 5h) and evaluated using flow cytometry (Fig. 5j). The ratio of PI + EC and annexin V + /PI + EC in the co-culture system significantly decreased when microglia was treated with BAY11-7082 after OGDR (Fig. 5i–k). Moreover, the ratio of p-RIP1/RIP1 and p-MLKL/MLKL in co-cultured EC also decreased in the NF-κB inhibitor treated group after OGDR (Fig. 5l–n), indicating that inhibition of microglia NF-κB nuclear translocation decreases endothelial necroptosis under OGDR.

### Infliximab blocks necroptosis of EC co-cultured with microglia under OGDR in vitro

**Fig. 6:**
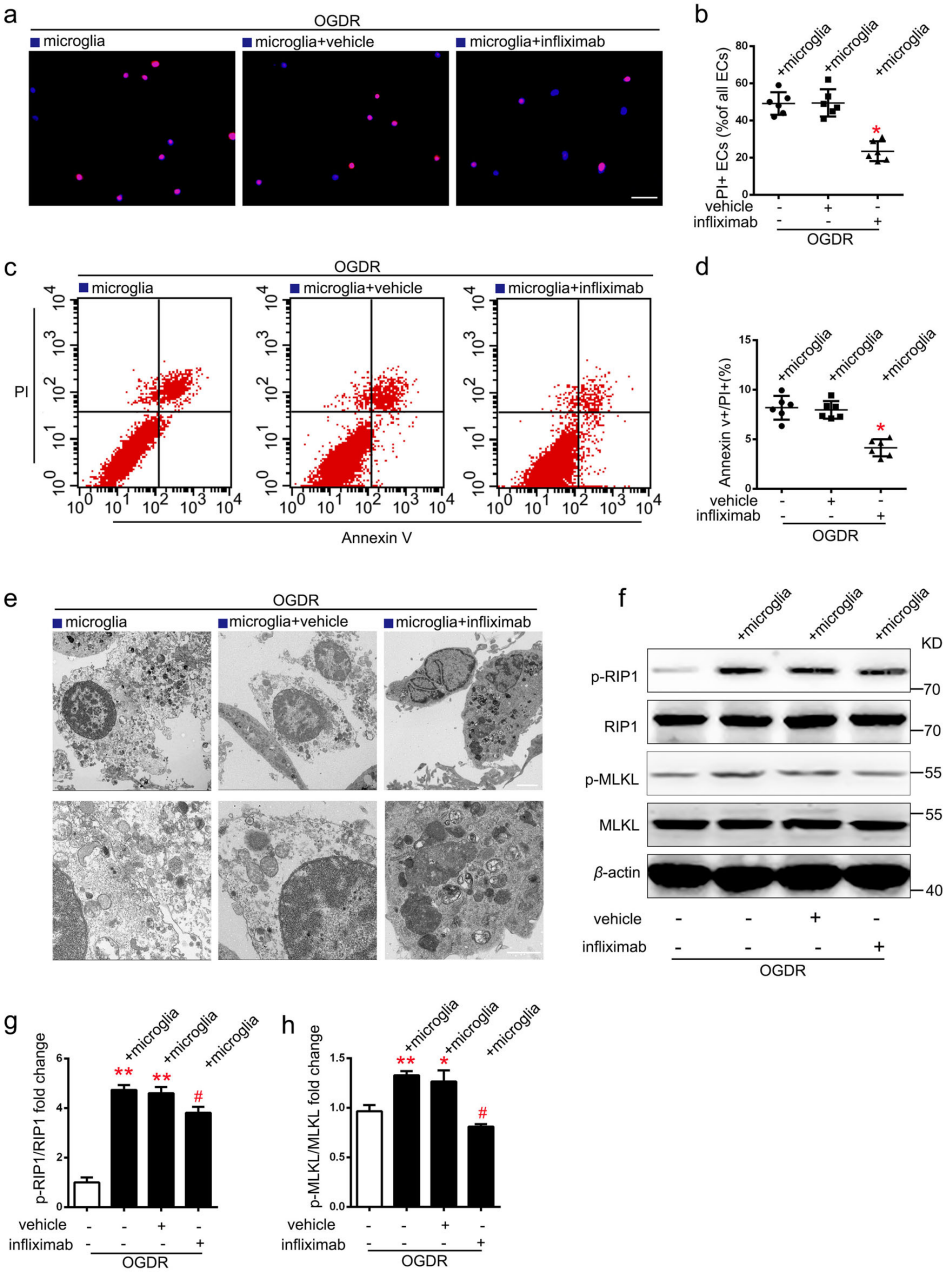
Infliximab reduces necroptosis of ECs co-cultured with microglia after OGDR. **a–d** Representative images and statistical results of PI (red)/Hoechst (blue) staining and PI/annexin V flow cytometry of ECs treated with or without infliximab (67 nM) in co-culture system under OGDR. *n* = 6; *P *<* 0.01 vs. vehicle group; Scale bar: 100 μm. **e** electron microscopy images of ECs show that co-cultured ECs treated with infliximab had relatively intact cytoplasmic membrane. *n* = 3; Scale bar: 2 μm (merged pictures) and 1 μm (magnified pictures). **f–h** Expression levels of p-RIP1/RIP1 and p-MLKL/MLKL in ECs treated with or without infliximab (67 nM) in co-culture system under OGDR. *n* = 3; *P < 0.05 vs. control group; **P < 0.01 vs. control group; ^#^*P* < 0.05 vs. vehicle group

Infliximab, a human and mouse chimeric monoclonal antibody that specifically blocks TNF-α, has been extensively used in clinic^22,23^. Addition of infliximab to the lower chamber (endothelial) of the co-culture system resulted in a significant reduction of the ratio of PI + /Hoechst + EC compared to that of the vehicle treated group under OGDR conditions (Fig. 6a, b). Flow cytometry results also agreed with these findings (Fig. 6c, d). Electron microscope analyses showed that, in the vehicle group, EC with mitochondrial swelling, translucent cytoplasm, enlarged cell volume, and plasma membrane disruption were present. In contrast, mitochondrial damage and cytoplasm transparency were significantly reduced by infliximab treatment (Fig. 6e). Similarly, the up-regulation of p-RIP1/RIP1 and p-MLKL/MLKL expression levels of EC in the co-culture system was inhibited by treatment with infliximab after OGDR (Fig. 6f–h). Migration tests showed that infliximab dramatically attenuated microglia migration under TNF-α condition (Supplementary Fig. 2c). These results suggest that blocking TNF-α signaling inhibits EC necroptosis induced by co-cultured microglia after OGDR.

### Infliximab reduces BBB permeability and improves stroke outcome after tMCAO

**Fig. 7:**
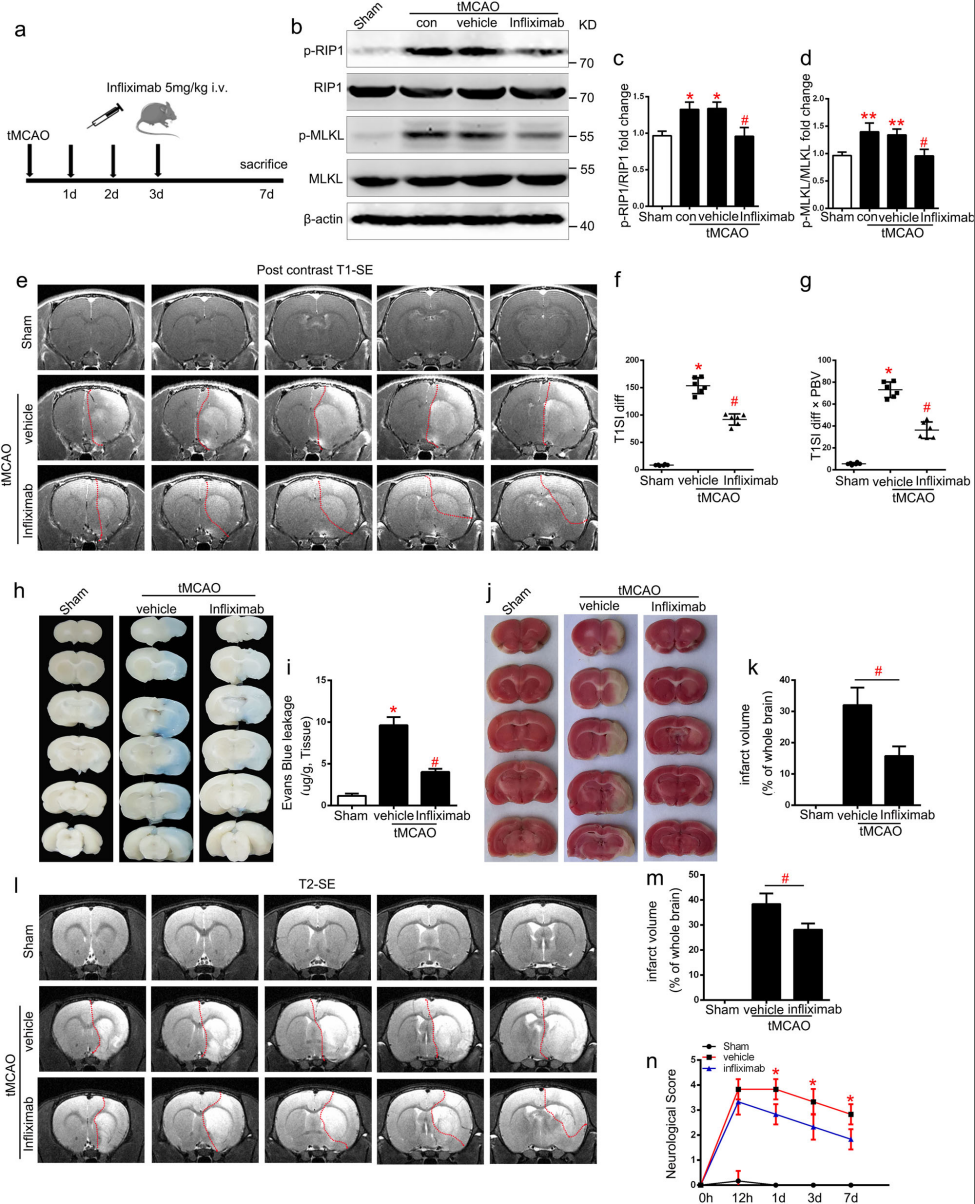
Infliximab provides protective effects against BBB disruption and I/R injury after stroke. **a** Experimental scheme. **b–d** Endogenous protein expression of p-RIP1/RIP1 and p-MLKL/MLKL in ischemic lateral cortex after tMCAO (3 day) from different groups. *n* = 6; *P *<* 0.05 vs. Sham group; **P *<* 0.01 vs. control group; ^#^*P* *<* 0.05 vs. vehicle group. **e–g** Representative MRI post-contrast T1-SE images and statistical BBB permeability represented by T1SI-diff and T1SI-diff × PBV of rat brains from each group. *n* = 6; *P *<* 0.001 vs. Sham group; ^#^*P* *<* 0.01 vs. vehicle group. **h**, **i** Evans blue leakage of rat brains in coronal sections (**h**) and extravasation (μg/g tissue) (**i**) from each group. *n* = 3; *P *<* 0.001 vs. Sham group; ^#^*P* *<* 0.01 vs. vehicle group. **j–m** Representative images and statistical results of TTC and MRI T2-SE imaging. *n* = 6 for MRI, n = 3 for TTC staining; ^#^*P* *<* 0.01 vs. vehicle group. **k**) Modified neurological severity score from day 0 to day 7 after tMCAO in different groups. *n* = 6; ^*^P *<* 0.05 vs. vehicle group

**Fig. 8:**
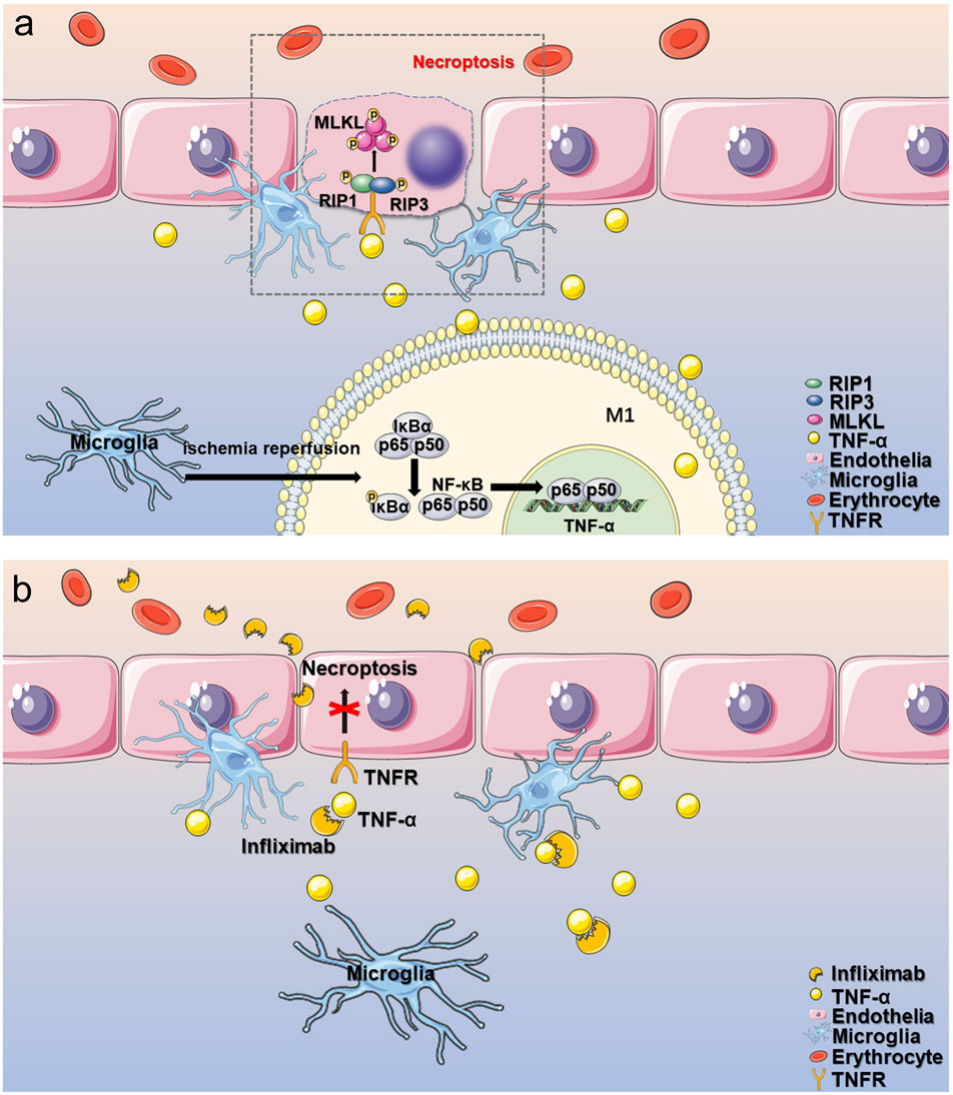
Schematic representation shows the role of infliximab in EC necroptosis after stroke. **a** After ischemic reperfusion, microglia cells are activated and transform into the M1 type, mediating phosphorylation and nuclear translocation of NF-kB and initiating TNF-α transcription. Secreted TNF-α binds to TNF receptor on ECs, activating necroptosis and leading to BBB disruption. **b** Infliximab, a specific antibody against TNF-α, blocks the binding of TNF-α with TNF receptor and inhibits the necroptotic signaling, alleviating BBB disruption

As the protective effects of infliximab in vitro were confirmed, we next explored the role of infliximab in vivo after tMCAO. After multiple *i.v*. doses (5 mg/kg) of infliximab injected 1 d, 2 day, and 3 day after reperfusion (Fig. 7a). Western blotting analysis showed that the up-regulation of p-RIP1/RIP1 and p-MLKL/MLKL expression levels in the periinfarct area were inhibited after treatment with infliximab after tMCAO (Fig. 7b–d).To further study the therapeutic effect of infliximab in tMCAO, rats were subjected to MRI, Evans blue extravasation assays, 2,3,5-Triphenyltetrazolium chloride (TTC) staining (3 day), and neurological severity score assessments (12 h, 1 day, 3 day, and 7 day). MRI postcontrast T1-SE sequencing (Fig. 7e) and Evans blue extravasation (Fig. 7h) results were used to evaluate BBB permeability. Quantification of results showed that infliximab ameliorated BBB disruption after stroke (Fig. 7f, g and i). In addition, the infarct volume was evaluated using TTC staining (Fig. 7j) and MRI T2-TSE sequencing (Fig. 7l). Quantification of results revealed a remarkable reduction of infarction volume compared with the vehicle group (Fig. 7k–m). Neurological severity score results revealed that multiple doses of infliximab improved neurological function on the 3rd day after tMCAO (Fig. 7n). These results demonstrate that infliximab is efficient and effective in protecting against BBB breakdown and I/R injuries after stroke (Fig. 8).

## Discussion

As the primary pathologic processes, EC death facilitates the destruction of BBB integrity and function^4,24^, a characteristic feature at the early stage after I/R injury, which is relevant to the poor clinical prognosis^2^. However, the precise mechanisms of EC death need further clarification. Apoptosis is a well-known, but not the only cause of EC loss in brain microvessels after stroke^25^. Here, we found for the first time that EC necroptosis might be an important cause leading to BBB disruption after ischemic stroke. Our study suggested that: (1) EC necroptosis was activated in ischemic penumbra after I/R injury; (2) EC necroptosis increased BBB permeability and ischemic brain injury; (3) M1-like microglial-derived TNF-α induced EC necroptosis; (4) NF-κB nuclear translocation increased the expression of TNF-α in M1-like microglia after I/R injury, and (5) Infliximab, a potent clinical drug blocks TNF-α, could ameliorate BBB disruption and stroke damage.

Necroptosis, an important programmed cell death mechanism, serves as a vital regulator in disease^26^. The key step under the necroptosis pathway induced by TNF-α is the kinase-regulated process of RIP1-RIP3-MLKL. The phosphorylation of these molecules not only promote to form the necrosome, but also trigger multiple cascades at the same time, mediating multiple pathological processes that leads to further damage^27,28^. More and more cell types under different pathophysiological microenvironment have been demonstrated to exist necroptosis, such as aging^29^, cancer (lung cancer metastasis)^10^, neurodegeneration (amyotrophic lateral sclerosis and Alzheimer’s disease)^9,30^ and autoimmune disease (multiple sclerosis)^11^. Furthermore, necroptosis has a pivotal role in many ischemic diseases. It has been reported that necroptosis involved in adverse cardiac remodeling after myocardial infarction^31^and neuron loss after ischemic stroke^14^. However, few studies concerned the role of necroptosis in mediating EC damage after I/R injury. Thus, it is valuable to identify the underlying mechanisms between necroptosis and EC loss, which may provide a promising and reasonable strategy for treating vascular injury and BBB disruption after cerebral I/R injury.

A pioneer study reported that necroptosis was first identified as a result of inflammation^8^. And the inflammatory microenvironment produced by microglial cells has been considered to participate in neuron injury and BBB disruption after stroke^32,33^. In addition, previous studies suggested that perivascular microglia contribute to BBB breakdown and disintegration in the ischemic penumbra^32^. However, the mechanism of activated microglia on vascular endothelial damage after I/R injury remains to be further explored. In this study, our results were consistent with the previous study^17^ that the number of perivascular microglia increased after tMCAO. And we found that the number of activated microglia contacting necroptotic EC in periinfarct area also increased after tMCAO. Additional studies indicated that the transform of polarity plays a pivotal role in microglial activation and inflammatory cytokines (such as TNF-α) release^34^. Here, we demonstrated that microglia can obtain a M1-like polarization and produce a large amount of TNF-α under OGDR. In addition, evidence from previous researches also suggested that microglia might be a major source of TNF-α in model of permanent MCAO^35^. In our current study, Iba1 and TNF-α double staining suggested that TNF-α was mainly expressed in microglia, which increased from 12 h and reached the highest levels on the 3rd day after tMCAO. Moreover, we also indicated that activated M1-like microglia could induce EC necroptosis in vitro.

In previous studies, proinflammatory cytokine in inflammatory-microenvironment, such as TNF-α, has been reported to be the trigger of necroptosis^36,37^. Consistently, most of proinflammatory mediators are derived from M1-like microglia, which is activated at the early stage after stroke^16^. Further study found that M1-like microglia could increase necroptosis of astrocytes in spinal cord injury^38^. In our current study, by knocking-down M1-like microglia-released iNOS, IL-6, IL-1β, and TNF-α, we identified TNF-α as a key proinflammatory cytokine that triggers EC necroptosis. However, although TNF-α has been shown to be the main promoter of endothelial necroptosis in this study, other pro-inflammatory cytokines released by M1-like microglia could be involved in the process of necroptosis, such as IFN-α^39^, which is remained to be further explored.

Recent work reported that inhibition of NF-κB signaling pathway depressed the production of many proinflammatory cytokines including TNF-α^34^. To explore the role of NF-κB in regulating macroglia-derived TNF-α production that mediates endothelial necroptosis, we used BAY11-7082 (an NF-κB inhibitor) to decrease the nuclear translocation of NF-κB, which not only suppressed TNF-α produce in microglia but also depressed the EC necroptosis in co-culture system. These data suggested that NF-κB may be essential in promoting microglial activation and TNF-α release, which mediates endothelial necroptosis, accelerates BBB disruption after I/R injury consequently.

There are few effective therapies in clinical management of ischemic stroke except for thrombolysis and mechanical thrombectomy, which are strictly time restricted after stroke^40^. Nec-1 is a well-known small-molecule inhibitor for necroptosis, which could alleviate many kinds of damage caused by necroptosis in animal experiment^41^. However, there is still lack of a safe and effective medicine for blocking necroptosis in clinic. We found that infliximab, a chimeric monoclonal antibody and a biological drug that works against TNF-α and is widely used to treat autoimmune diseases, such as inflammatory bowel disease and multiple sclerosis^42,43^, efficiently blocks the necroptosis of EC in vitro and in vivo. Therefore, our study expanded indications of this medicine to BBB protection and neurological functions improvement after ischemic stroke.

In summary, our study shows for the first time a previously unexplored role of M1-like microglia-derived TNF-α mediated EC necroptosis in BBB disruption after ischemic stroke. Importantly, this study about endothelial necroptosis also sheds new light on a novel therapeutic target for stroke, and infliximab is suggested to be a potential drug for stroke treatment.

## Materials and methods

### Establishment of tMCAO model

All rat experiments were approved by the Medical Ethics Committee of Tongji Medical College and the Institutional Committee of Animal Care and Use, Huazhong University of Science and Technology (HUST), Wuhan, China. Male Sprague-Dawley (SD) rats, weighing 250–300 g, were anesthetized with pentobarbital (40–60 mg/kg, i.p.) and the tMCAO model was established as described^44^. Intracerebroventricular injections was performed as previously reported^45,46^. Drugs were injected into the right lateral ventricle at 1.0 mm posteriorly to bregma, 2.5 mm laterally from midline, and 3.5 mm vertically from the skull surface. Neurological function assessment was performed 1–7 day after tMCAO using the modified 7-point neurological severity score scale as previously described^47^. TTC (2%) staining was performed to confirm the infarction. MRI detection, Evans blue extravasation, and brain sampling were conducted.

### MRI detection

MRI detection was performed on a Bruker BioSpec 7T/20 scanner (Ettlingen, Germany) as previously described^48^. The extent of BBB leakage was represented by T1-spin echo (T1-SE) images (precontrast and postcontrast). Firstly, T1-SE (precontrast) and T2-TSE images were acquired before intravenous injection of gadolinium-diethylenetriamine pentaacetic acid (Gd-DTPA) (0.2 mM/kg). Secondly, postcontrast T1-SE images (Gd-DTPA permeable BBB volume (PBV)) were acquired 30 min after injection. Average pixel intensity of hyperintense (T1SI-diff) subtracted from pre- and postcontrast T1-SE images was calculated. The product of PBV and T1SI-diff represented the permeability change of BBB^49^. Higher-intensity volume on T2-TSE images represented ischemic volume. Infarct volume was calculated as previously described^50^.

### Evans blue extravasation

Evans blue extravasation was performed for evaluating BBB integrity as previously described^51^. Briefly, rats were anesthetized 6 h after Evans blue (4% in saline solution, 2 mL/kg; Sigma Aldrich, St. Louis, MO, USA) intravenous administration, and then transcardially perfused with PBS. Randomly chosen rats continued to be perfused with 4% paraformaldehyde (PFA) and then those brains were cut into five sections each. The remaining brains were separated into left and right hemispheres respectively, weighed, homogenized with 1 mL of 50% trichloroacetic acid, and centrifuged (14,000*×g*) for 30 min. Then, the supernatant was collected and mixed with absolute ethanol (1:3). Evans blue concentration (mg/g tissue) determined by absorbance at 630 nm with spectrophotometry represented BBB permeability.

### PI/TUNEL staining

PI/TUNEL staining was performed as previously reported^52^. Briefly, PI was intraperitoneally injected (1 μg/g; Sigma-Aldrich, St. Louis, MO, USA) while the tMCAO model was established. Cell apoptosis was detected via TUNEL (Roche, Mannheim, Germany) staining. Frozen brain sections were incubated with TUNEL (37 °C for 1 h), washed three times with PBST (PBS with 0.5 ‰ Tween-20), and then mounted and visualized with a Nikon A1-Si confocal microscope (Nikon, Tokyo, Japan). As reported previously, PI−/TUNEL+ and PI+/TUNEL+ indicate apoptosis and necrosis, respectively^53^.

### Immunofluorescent staining

Immunofluorescent staining was performed as described^47,54^. All rats were transcardially perfused with PBS, then brain tissues were fixed with 4% PFA, and frozen blocks were cut into 8–20 μm sections. The following primary antibodies were used: p-MLKL (Rabbit, 1:50, ab187091; Abcam, UK), Iba1 (Mouse, 1:50, ab15690 and Goat, 1:50, ab5076; Abcam, UK), CD31 (Mouse, 1:50, ab119339; Abcam, UK), and TNF-α (Rabbit, 1:100, ab6671; Abcam, UK). The secondary antibodies are shown in Supplementary Table 1. Nuclei were stained with 4′,6-diamidino-2-phenylindole (DAPI, Invitrogen). Samples were visualized with a Nikon A1Si confocal microscope (Nikon, Japan). Co-localization and percentage-positive area images were analyzed and performed using NIS Elements AR Imaging Software 4.10 (Nikon) and ImageJ 1.41 software.

### Electron microscopy

Electron microscopy was performed as previously reported^55^. After a quick wash with cold PBS, brain samples or Endothelium were fixed in fixation fluid (2.5% glutaraldehyde and 0.5% osmium tetroxide), dehydrated, and embedded with Spurr’s epoxy resin. Ultrathin sections (90 nm) were made and stained with uranyl acetate and lead citrate, which viewed by a JEOL JEM-1010 transmission electron microscope.

### Primary culture of rat brain microvascular EC and microglia cells

Rat brain microvascular EC were isolated from SD rats (3–5-weeks-old) as previously described, with some modifications^56^. Rat cerebral cortices were minced into small pieces, then digested in DMEM (Hyclone) with 1000 U/mL DNase I and 0.1% collagenase II at 37 °C for 40 min before centrifugation at 500 *×* *g* for 5 min at 4 °C. The precipitate was resuspended in DMEM with 17% Percoll (Amersham Pharmacia Biotech, Piscataway, NJ, USA) and centrifuged at 2500 *×* *g* for 10 min at 4 °C, after that, the precipitate on the bottom was resuspended in DMEM with 33% Percoll and for gradient centrifugation centrifuged at 2500 *×* *g* for 10 min at 4 °C. Purified microvessels were harvested from the middle layer suspended matter and washed with ice-cold PBS twice. Microvessels were then seeded into 6-well plates with EC medium (#1001, ScienCell Research Laboratories, San Diego, California, USA), incubated at 37 °C in humidified 5% CO_2_. After three days, ECs were plated in six-well plates (1–1.5 × 10^6^ per plate) or 12-well plates (3–4 × 10^5^ per plate) with fresh medium.

Primary microglia cells were prepared from primary mixed glial cell cultures as previously reported^55,57^. Briefly, neonatal rat cerebral cortices were minced into small pieces and digested in DMEM with 0.25 mg/mL trypsin and 1000 U/mL DNase at 37 °C for 20 min before centrifugation at 500*×g* for 5 min at 4 °C. The precipitate was resuspended in DMEM with 10% fetal bovine serum (Hyclone, Logan, UT) with 100 U penicillin/100 μg streptomycin (Invitrogen). Mixed glia cells were then seeded into six-well plates and incubated at 37 °C in humidified 5% CO_2_ for 12–14 days till the mixed glial cultures reached confluency. Next, microglia cells were separated from the mixed glial cells by shaking the flasks at 200 r.p.m. for 4 h and collected by centrifugation. Finally, microglia cells were plated in six-well plates (1–1.5 × 10^6^ per plate) or 12-well plates (3–4 × 10^5^ per plate) with fresh medium.

### Acute isolation of microglia after tMCAO

All rats were anesthetized and transcardially perfused with PBS. lateral cortex tissues of peri-infarct area at different time point after tMCAO were dissected, digested with Liberase TL (1.6 WÃ¼nsch/ml, Sigma, cat: 540102001) and DNAse I (Sigma, cat: DN25) at 37 °C for 1 h, microglia were isolated by Percoll gradient centrifugation as previously described^58,59^.

### OGDR treatment

Cells were cultured with glucose-free medium in a hypoxic chamber (Thermo Fisher Scientific, USA, 1% O_2_, 94% N_2_, and 5% CO_2_) at 37 °C for 2 h. Then cells were returned to normal cell culture incubators (95% air and 5% CO_2_) with normal medium (containing 5.5 mM glucose) for 2–12 h. Control cells were incubated in normal medium containing 5.5 mM glucose under normal culture conditions for the same time period^60^.

### Small interfering RNA transfection

Microglial cells were transfected with 10 nM siRNA against TNF-α, IL-1β, iNOS, and IL-6, or 10 nM control siRNA (purchased from GuangZhou RIBOBIO) for 24 h by using Lipofectamine 2000 transfection reagent (Thermo Fisher Scientific, USA) following the manufacturer’s instructions.

### PI/Hoechst staining assay

Cell death was evaluated with PI and Hoechst staining according to the manufacturer’s instructions. Cultured cells were stained with Hoechst 33258 (5 μL/mL, Beyotime, Shanghai, China) for 10 min at 4 °C and PI (5 μL/mL, Beyotime, Shanghai, China) for 15 min at 37 °C. Then cells were fixed with 4% PFA for 15 min at room temperature. Stained samples were analyzed by a fluorescence microscope (Olympus IX73, Japan).

### Flow cytometry

Cell death was assessed using flow cytometry with an assay kit (556547, BD Biosciences Pharmingen, San Diego, CA, USA) as described^61^. Briefly, ECs in different groups were trypsinized with 0.25 % trypsin (without EDTA), and 1 × 10^6^ cells were counted and washed in ice-cold PBS twice. After centrifugation at 2500 r.p.m. for 5 min, cells were resuspended in 200 uL binding buffer. After sequential staining with 5 μL FITC-conjugated annexin V and 5 μL PI, cells were analyzed using a flow cytometer. Data was analyzed using FlowJo software (Treestar, Ashland, OR, USA).

### Real-time quantitative PCR

Total RNA was extracted from microglia under different treatments using TRIzol reagent (Takara, Kyoto, Japan). Samples were reverse transcribed using a cDNA Synthesis Kit (Takara, Kyoto, Japan). Then, cDNA was amplified by a SYBR Premix Ex Taq kit (Takara, Kyoto, Japan). Primers were shown in Supplementary Table 2. All procedures were performed according to the manufacturers’ instructions. Data were analyzed using the comparative Ct method (2^−ΔΔCt^), normalized to β-actin, and expressed as fold-change compared to the control.

### Western blotting analysis

Western blotting analysis was performed as previously reported^62^. An equal amount (40–80 μg) of protein samples were detected. Primary antibodies against p-MLKL (Rabbit, 1:500, ab187091; Abcam, UK), MLKL (Rabbit 1:1000, ab194699; Abcam, UK), p-RIP1 (Rabbit, 1:500, 65746; Cell Signaling Technology, USA), RIP1 (Mouse, 1:500, ab72139; Abcam, UK), caspase 8 (Rabbit, 1:1000, A0215; ABclonal, China), caspase 3 (Rabbit, 1:1000, A11319; ABclonal, China), TNF-α (Rabbit, 1:500, ab6671; Abcam, UK), IL-6 (Mouse, 1:500, ab9324; Abcam, UK), iNOS (Rabbit, 1:500, ab15323; Abcam, UK), IL-1β (Rabbit, 1:500, ab2105; Abcam, UK), NF-kB p65 (Rabbit, 1:1000; ab16502, Abcam, UK), IkBα (Mouse, 1:1000, 4814; Cell Signaling Technology, USA), p-IkBα (Rabbit, 1:1000, 2859; Cell Signaling Technology, USA); H3 (Rabbit, 1:1000, AF0009; Beyotime, China) and β-actin (Rabbit, 1:3000, AC026; ABclonal, China) were used. Protein expression levels were analyzed using ImageJ software and normalized to β-actin (National Institutes of Health, Bethesda, MD, USA). Phosphorylated protein expression was evaluated compared to total protein expression.

### ELISA assay

TNF-α expression in culture supernatants were detected using a TNF-α ELISA KIT (eBioScience) according to the manufacturer’s instruction. Absorption at 450 nm was determined with a microplate reader (Bio-Rad, iMark, USA), and TNF-α concentration was determined according to the standard curve generated at the same time.

### Drug treatment

Nec-1 (MedChem Express, St. Louis, MO, USA, HY-15760) was dissolved in DMSO and injected (1 μg) into the lateral ventricles of ischemic cortex after establishment of tMCAO. BAY11–7082, a NF-kB p65 inhibitor (MedChem Express, St. Louis, MO, USA), was dissolved in DMSO to a concentration of 10 mM (1000x stock solution) and added to microglia 30 min before OGDR treatment^63^. Infliximab, a monoclonal antibody that blocks TNF-α, was dissolved in PBS (10 mg/mL) and administered through tail vein injection (5 mg/kg) at 1 day, 2 days, and 3 days after reperfusion in vivo and added to Endothelium (67 nM) under OGDR^64,65^ in vitro. The vehicle group received the same volume of solvent with the same administrative methods.

### Statistical analysis

All data are shown as mean ± S.E.M. (*n* ≥ 3). Statistical analyses were performed using GraphPad Prism 5.0 software (GraphPad, San Diego, CA, USA). The normality of distribution was analyzed by A Shapiro-Wilk test. Comparisons between two groups were performed using a Student t test (Figs. 1l, 2b–n, 3f–k, 4g–l, 5f, g, 7f–m, and s2c). One-way ANOVA was used to statistical analysis among more than two groups (Figs. 1b, d, 1f–i, 3b, 4a–f, 4n–p, 5b, d, i, k, m, n, 6b, d, 6g–h, 7c–d, n, s1a, and s2e). *P* < 0.05 was considered to be statistically significant.

## Supplementary information

Information of secondary antibodies used for Immunofluorescent staining

Information of primers used for Real-time quantitative PCR

EC necroptosis does not increase under OGDR condition

Infliximab attenuated TNF-α-induced microglia migration

Negative controls of immunofluorescent staining of rat brain sections

Supplementary figure legends
