# Supplementary material for: Microglia-derived TNF-α mediates endothelial necroptosis aggravating blood brain–barrier disruption after ischemic stroke
Source: Cell Death Dis. 2019 Jun 20;10(7):487. doi: 10.1038/s41419-019-1716-9 (PMC6586814; doi:10.1038/s41419-019-1716-9)
Supplement: Supplementary file 1 — Information of secondary antibodies used for Immunofluorescent staining [file 41419_2019_1716_MOESM1_ESM.docx]

| Donkey anti-Mouse IgG | Alexa Fluor 488 | A21206 | Thermo Fisher Scientific, USA |
| --- | --- | --- | --- |
| Donkey anti-Mouse IgG | Alexa Fluor 594 | A21203 | Thermo Fisher Scientific, USA |
| Goat anti-Rabbit IgG | Alexa Fluor 488 | A11008 | Thermo Fisher Scientific, USA |
| Goat anti-Rabbit IgG | Alexa Fluor 594 | A32740 | Thermo Fisher Scientific, USA |
| Donkey anti-Goat IgG | Alexa Fluor 594 | A32758 | Thermo Fisher Scientific, USA |
| Alexa Fluor 647 Donkey anti Goat IgG |  | ANT033 | antgene, China |

**Supplementary table 1.** **Information of secondary antibodies used for Immunofluorescent staining**
