# Supplementary material for: Microglia-derived TNF-α mediates endothelial necroptosis aggravating blood brain–barrier disruption after ischemic stroke
Source: Cell Death Dis. 2019 Jun 20;10(7):487. doi: 10.1038/s41419-019-1716-9 (PMC6586814; doi:10.1038/s41419-019-1716-9)
Supplement: Supplementary file 2 — Information of primers used for Real-time quantitative PCR [file 41419_2019_1716_MOESM2_ESM.docx]

| TNF-α-Rat-forward | 5’- GCATGATCCGAGATGTGGAACTGG-3’ |
| --- | --- |
| TNF-α-Rat-reverse | 5’-CGCCACGAGCAGGAATGAGAAG-3’ |
| IL-1β-Rat-forward | 5’-ATCTCACAGCAGCATCTCGACAAG-3’ |
| IL-1β-Rat-reverse | 5’-CACACTAGCAGGTCGTCATCATCC-3’ |
| IL-6-Rat-forward | 5’-AGGAGTGGCTAAGGACCAAGACC-3’ |
| IL-6-Rat-reverse | 5’-TGCCGAGTAGACCTCATAGTGACC-3’ |
| iNOS-Rat-forward | 5’-GAGACGCACAGGCAGAGGTTG-3’ |
| iNOS-Rat-reverse | 5’-CAGGAAGGCAGCAGGCACAC-3’ |
| CD206-Rat-forward | 5’-CCTATGAAAATTGGGCTTACGG-3’ |
| CD206-Rat-reverse | 5’-CTGACAAATCCAGTTGTTGAGG-3’ |
| Arg1-Rat-forward | 5’-GCCTGAGAGTCTGGCCTA CA-3’ |
| Arg1-Rat-reverse | 5’-ATCCCTCAGGCTGCTCCATT-3’ |
| β-actin-Rat-forward | 5’-AGACTTCGAGCAGGAGATGG-3’ |
| β-actin-Rat-reverse | 5’-CCATCATGAAGTGTGACGTTG-3’ |

**Supplementary table 2. Information of primers used for Real-time quantitative PCR**
