# Supplementary figures and images for: Microglia-derived TNF-α mediates endothelial necroptosis aggravating blood brain–barrier disruption after ischemic stroke
Source: Cell Death Dis. 2019 Jun 20;10(7):487. doi: 10.1038/s41419-019-1716-9 (PMC6586814; doi:10.1038/s41419-019-1716-9)

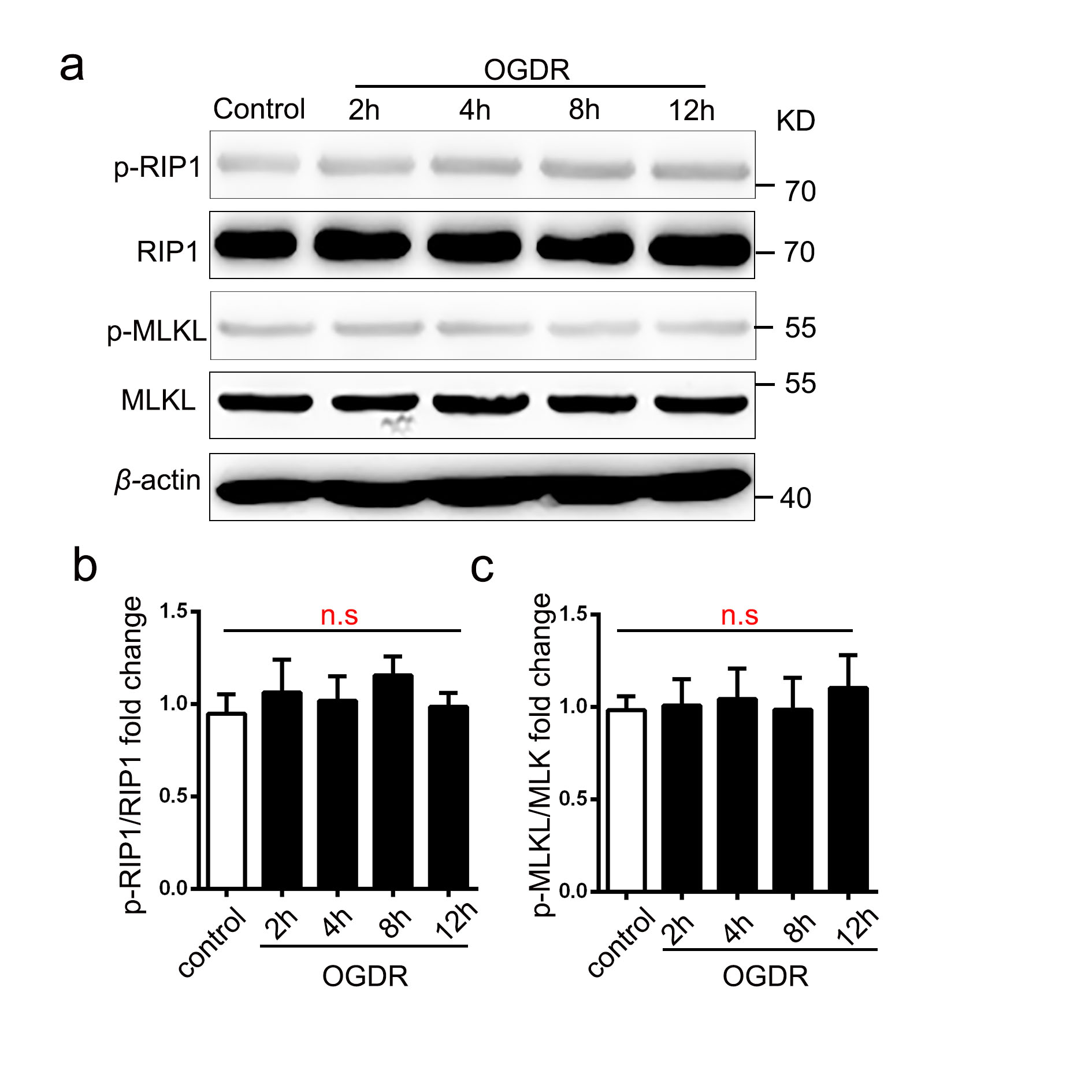

Supplement: Supplementary file 3 — EC necroptosis does not increase under OGDR condition [file 41419_2019_1716_MOESM3_ESM.jpg]

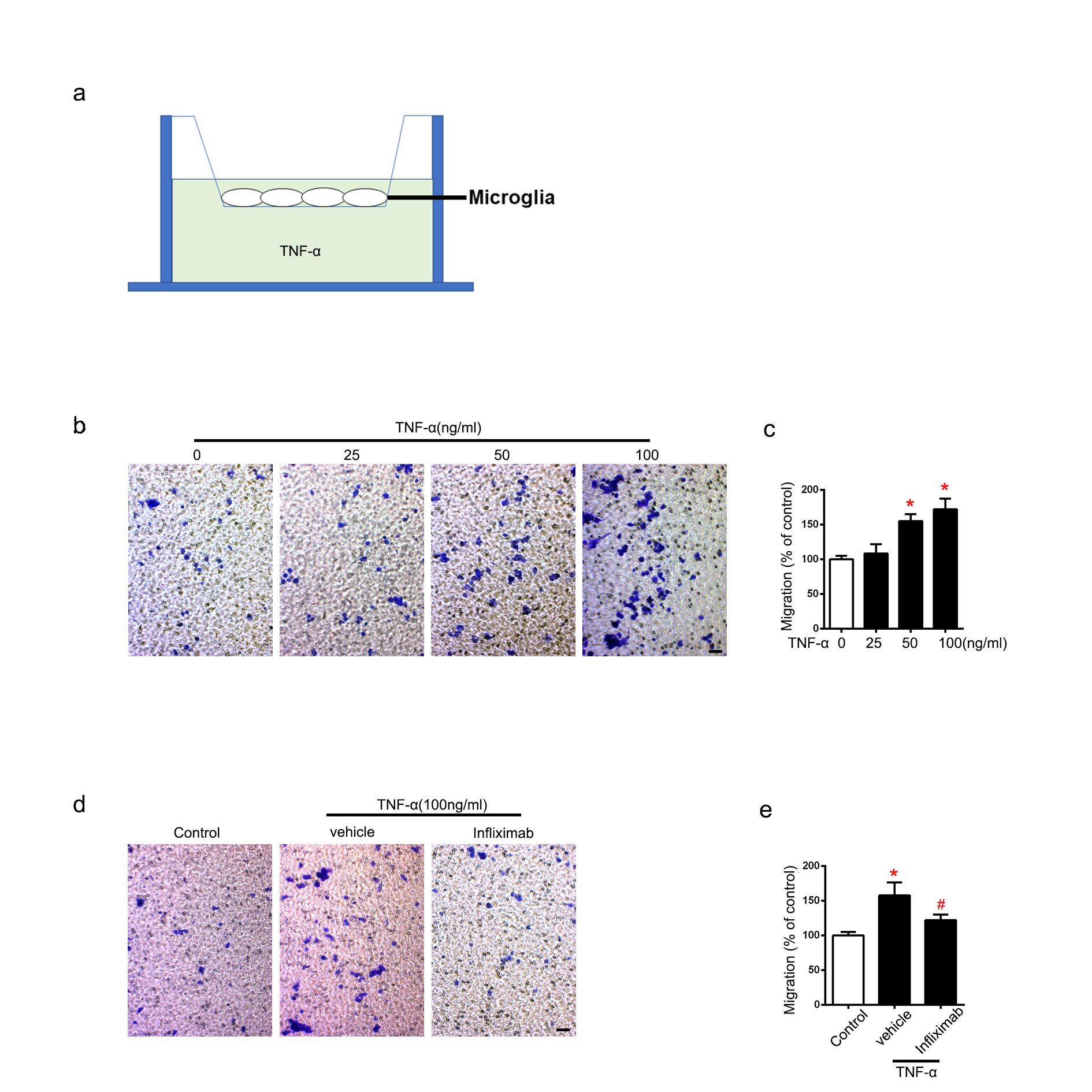

Supplement: Supplementary file 4 — Infliximab attenuated TNF-α-induced microglia migration [file 41419_2019_1716_MOESM4_ESM.jpg]

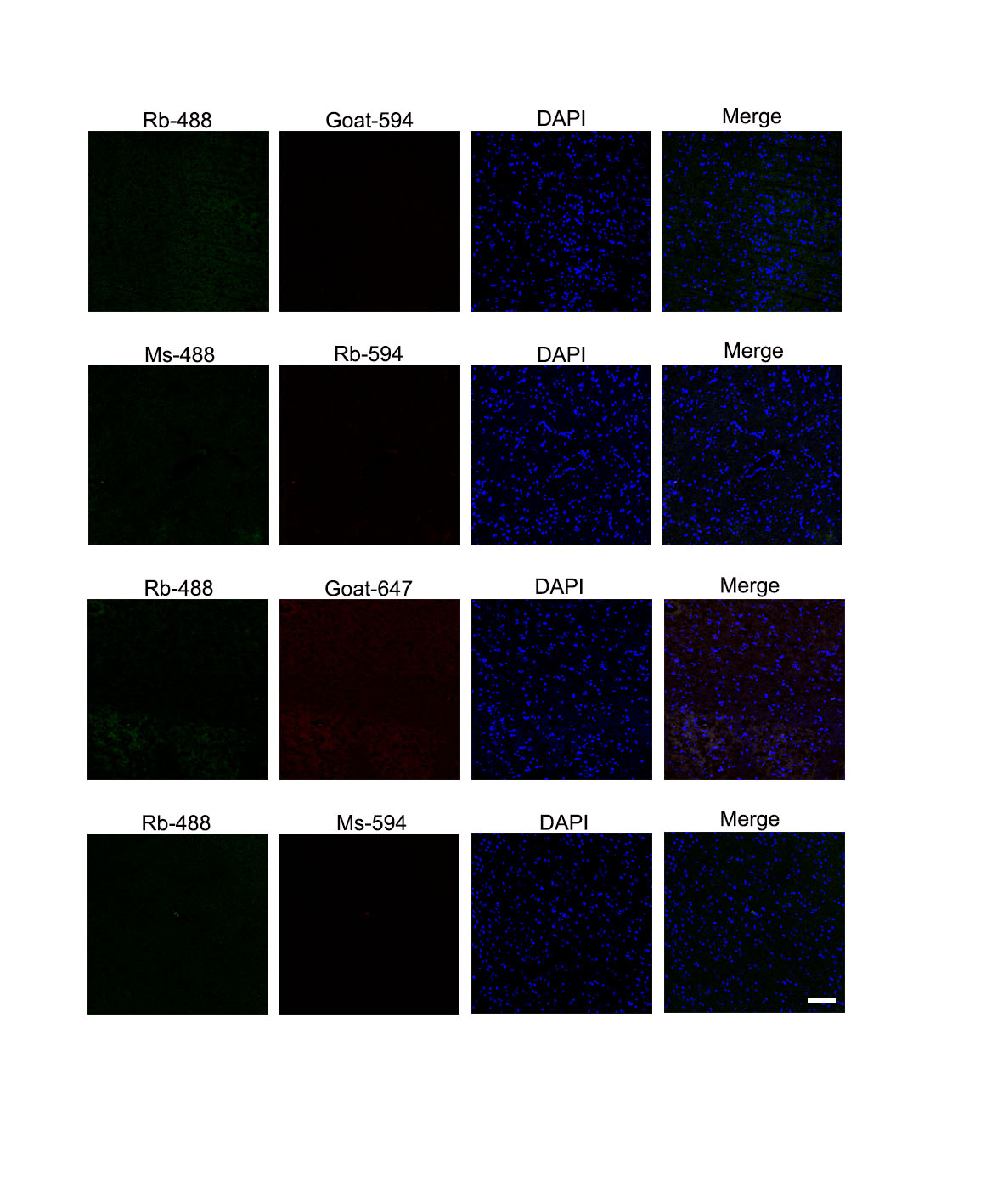

Supplement: Supplementary file 5 — Negative controls of immunofluorescent staining of rat brain sections [file 41419_2019_1716_MOESM5_ESM.jpg]
