# Supplementary material for: Microglia-derived TNF-α mediates endothelial necroptosis aggravating blood brain–barrier disruption after ischemic stroke
Source: Cell Death Dis. 2019 Jun 20;10(7):487. doi: 10.1038/s41419-019-1716-9 (PMC6586814; doi:10.1038/s41419-019-1716-9)
Supplement: Supplementary file 6 — Supplementary figure legends [file 41419_2019_1716_MOESM6_ESM.docx]

**Supplementary Figure 1. EC necroptosis does not increase under OGDR condition**

a-c) Expression of p-RIP1/RIP1 and p-MLKL/MLKL in ECs subjected to OGDR for 2, 4, 8, and 12 h. n = 6; *P* > 0.05 *vs.* control group.

**Supplementary Figure 2.** **Infliximab attenuated TNF-α-induced microglia migration**

a) Illustration of microglial transwell migration assay. b-e) TNF-α-induced microglial migration in a dose-dependent manner (b-c) and Infliximab attenuated TNF-α-induced microglia migration (d-e). Scale bar: 50 μm; n = 3; **P* < 0.01 *vs.* Sham group; ^#^*P* < 0.05 *vs.* vehicle group.

**Supplementary Figure 3. Negative controls of immunofluorescent staining of rat brain sections**

a) Two-two combinations of secondary antibodies were used as negative controls. Nuclei were stained by DAPI (blue).

**Supplementary table 1.** **Information of secondary antibodies used for Immunofluorescent staining**

**Supplementary table 2.** **Information of primers used for Real-time quantitative PCR**
